# Indirect effects of the covid-19 pandemic on childhood infection in England: population based observational study

**DOI:** 10.1136/bmj-2021-067519

**Published:** 2022-01-12

**Authors:** Seilesh Kadambari, Raphael Goldacre, Eva Morris, Michael J Goldacre, Andrew J Pollard

**Affiliations:** 1 Oxford Vaccine Group, Department of Paediatrics, University of Oxford, Oxford, UK; 2 NIHR Oxford Biomedical Research Centre, Oxford, UK; 3 Unit of Health-Care Epidemiology, Big Data Institute, Nuffield Department of Population Health, University of Oxford, Oxford, UK

## Abstract

**Objective:**

To assess the impact of the covid-19 pandemic on hospital admission rates and mortality outcomes for childhood respiratory infections, severe invasive infections, and vaccine preventable disease in England.

**Design:**

Population based observational study of 19 common childhood respiratory, severe invasive, and vaccine preventable infections, comparing hospital admission rates and mortality outcomes before and after the onset of the pandemic in England.

**Setting:**

Hospital admission data from every NHS hospital in England from 1 March 2017 to 30 June 2021 with record linkage to national mortality data.

**Population:**

Children aged 0-14 years admitted to an NHS hospital with a selected childhood infection from 1 March 2017 to 30 June 2021.

**Main outcome measures:**

For each infection, numbers of hospital admissions every month from 1 March 2017 to 30 June 2021, percentage changes in the number of hospital admissions before and after 1 March 2020, and adjusted odds ratios to compare 60 day case fatality outcomes before and after 1 March 2020.

**Results:**

After 1 March 2020, substantial and sustained reductions in hospital admissions were found for all but one of the 19 infective conditions studied. Among the respiratory infections, the greatest percentage reductions were for influenza (mean annual number admitted between 1 March 2017 and 29 February 2020 was 5379 and number of children admitted from 1 March 2020 to 28 February 2021 was 304, 94% reduction, 95% confidence interval 89% to 97%), and bronchiolitis (from 51 655 to 9423, 82% reduction, 95% confidence interval 79% to 84%). Among the severe invasive infections, the greatest reduction was for meningitis (50% reduction, 47% to 52%). For the vaccine preventable infections, reductions ranged from 53% (32% to 68%) for mumps to 90% (80% to 95%) for measles. Reductions were seen across all demographic subgroups and in children with underlying comorbidities. Corresponding decreases were also found for the absolute numbers of 60 day case fatalities, although the proportion of children admitted for pneumonia who died within 60 days increased (age-sex adjusted odds ratio 1.71, 95% confidence interval 1.43 to 2.05). More recent data indicate that some respiratory infections increased to higher levels than usual after May 2021.

**Conclusions:**

During the covid-19 pandemic, a range of behavioural changes (adoption of non-pharmacological interventions) and societal strategies (school closures, lockdowns, and restricted travel) were used to reduce transmission of SARS-CoV-2, which also reduced admissions for common and severe childhood infections. Continued monitoring of these infections is required as social restrictions evolve.

**Figure:**
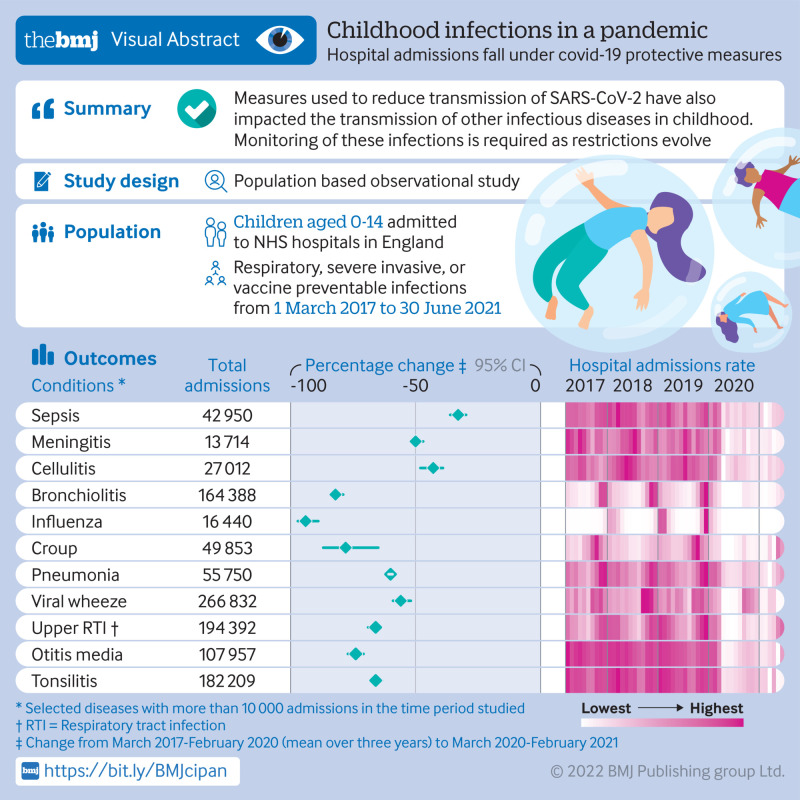


## Introduction

Children younger than 16 years have accounted for less than 2% of all people with covid-19.^1^ ^2^ ^3^ One of the earliest and largest epidemiological studies in children with symptomatic covid-19 showed that only 13 of 2143 (0.6%) children had developed critical illness.^4^ In Europe and North America, less than 1% of children admitted to hospital with covid-19 have died.^5^ ^6^

The indirect effects of covid-19 on children’s health appear to be substantial. Delayed presentations to the paediatric emergency department because of disruption in healthcare services, caregiver anxiety in being exposed to SARS-CoV-2 in a healthcare setting, and inconsistent public health messaging have been reported.^7^ ^8^ ^9^ Additionally, routine childhood immunisation programmes have been interrupted during the pandemic. In May 2020, the World Health Organization reported that services had been suspended or postponed in 68 lower income countries, affecting more than 80 million children younger than 1 year. This disruption is partly because of a lack of transport of vaccines between countries and a shortage of healthcare staff.^10^ In spring 2020, 22% of infants in the WHO European region had their vaccination courses interrupted.^11^ The first UK national lockdown began in March 2020, after which in London there was a reported 20% reduction in measles, mumps, and rubella vaccination counts,^12^ although national coverage of most routine childhood immunisations subsequently increased during 2020.^13^ Monitoring the rates of vaccine preventable disease, with disruptions to vaccinations and as herd immunity potentially wanes, is critical to understanding how to adapt immunisation programmes during the pandemic and achieve high rates of coverage. In addition to delayed presentations, attendances to paediatric emergency departments in countries worst affected by covid-19 have reduced considerably since the onset of the pandemic.^8^ ^14^ ^15^ During 2020, laboratory surveillance data and observational studies worldwide have shown major decreases in some childhood infections, which might have contributed to this reduction.^15^ ^16^ ^17^ ^18^ ^19^

Robust population based data are required to quantify and evaluate paediatric infection related admissions to hospital during the pandemic and their outcomes to provide evidence based public health messaging and policy implementation. The primary objective of this study was to assess the indirect effects of the covid-19 pandemic on hospital admissions for childhood infections in England. Specifically, this study reports on numbers of hospital admissions in England for 19 different respiratory infections, severe invasive infections, and vaccine preventable diseases in the 12 months from 1 March 2020 compared with the previous three years. Admissions with a diagnosis of covid-19 are also reported for reference. Our hypothesis was that the burden of childhood infections would be lower during the covid-19 pandemic because of a range of behavioural or societal measures that were implemented, but that an increase in vaccine preventable infections might occur. Future analyses will continue to track patterns of care until the challenges associated with the pandemic diminish.

## Methods

### Data sources

All episodes of care for children younger than 15 years admitted to NHS hospitals in England from 1 March 2017 to 30 June 2021 with the specified infections defined using ICD-10 (international classification of diseases, 10th revision; table 1) were extracted from a national dataset of Hospital Episode Statistics Admitted Patient Care records (HES) collected and supplied by NHS Digital. These records were linked to national mortality records collected by the Office for National Statistics (see linkage methods at https://digital.nhs.uk/data-and-information/data-tools-and-services/data-services/linked-hes-ons-mortality-data). Comprehensive timelines outlining the UK government response, through laws and lockdowns, can be found in a report published by the House of Commons library.^20^

**Table 1:** ICD-10 (international classification of diseases, 10th revision) codes for 19 infections, covid-19, and comorbidities included in the study

Diagnosis	ICD-10 codes
**Infection category**	
Severe invasive	
Sepsis	A40.0, A40.1, A41
Meningitis	A17.0, A17.8, A32.1, A85.1, A86, A87, B00.3, B00.4, B01.0, G01, G00.3, G00.8, G00.9, G02.0, G02.1, G03, G04
Septic arthritis	M00, M46.5
Osteomyelitis	M86
Pyelonephritis	N10, N16.0
Cellulitis	L03
Respiratory	
Bronchiolitis	J21.0, J21.1, J21.8, J21.9
Influenza	J10, J11
Croup	J05, J20.9
Pneumonia	J12.0, J12.2, J12.8, J12.9, J13, J14, J15.0, J15.1, J15.2, J15.4, J15.5, J15.6, J15.7, J15.8, J15.9, J16.0, J17, J18, P23
Viral wheeze	R06.2
Upper respiratory tract infection	J06
Otitis media	H65, H66, H67
Tonsilitis	J03, J36
Vaccine preventable	
* Neisseria meningitidis*	A39
* Haemophilus influenzae*	G00.0, A41.3
* Streptococcus pneumoniae*	G00.1, A40.3
Measles	B05
Mumps	B26
**Comorbidities**	
Asthma	J45
Bronchopulmonary dysplasia	P27.1
Extreme prematurity (<28 weeks)	P07.2 (or ‘gestat’ 22-27 wks)
Cystic fibrosis	E84
Congenital cardiac disease	Q20, Q21, Q24
Bronchiectasis	J47
Immunodeficiency with predominantly antibody defects	D80
Combined immunodeficiencies	D81
Common variable immunodeficiency	D83
Acute lymphoblastic leukaemia	C91.0
Acute myeloid leukaemia	C92.0
**Covid-19 **	U07.1, U07.2

### Study design

This is a population based observational study of children aged 0-14 in England. Supplementary table 1 shows the size of the population (population counts for 2021 were not available). Monthly counts of admissions in children aged 0-14 with each infection were determined from 1 March 2017 to 30 June 2021. These counts were aggregated into four 12 month periods from March 2017 to February 2021; the numbers of admissions in the 12 months from 1 March 2020 to 28 February 2021 were compared with the mean annual numbers from the preceding three years. From March 2021 onwards, comparisons were made on a monthly basis; that is, the numbers of admissions in each month in 2021 were compared with the mean number of admissions in the equivalent month in the three years from 2017 to 2019. Subgroup analyses were conducted by sex, age, geographical region, index of multiple deprivation,^21^ ethnic group, and by the comorbidities listed in table 1 (which were obtained by searching the patients’ hospital admission records from birth). For conditions where admission numbers were sufficiently high, the consistency of findings across individual NHS hospital trusts was investigated. Admissions that were followed by a recorded date of death within 60 days were counted as 60 day case fatalities; these rates were then compared before and after 1 March 2020.

### Statistical analysis

For each infective condition, the percentage change from the mean annual count in 2017-20 to the annual count in 2020-21 was calculated with 95% confidence intervals, assuming a negative binomial distribution. From March 2021 onwards, these comparisons were undertaken on a monthly basis using the same methods. To check the consistency of findings across individual NHS hospital trusts, boxplots were plotted to show the spread of the percentage changes in each hospital trust. Sixty day all cause case fatality rates (expressed per 10 000 people admitted) with 95% exact Clopper-Pearson binomial confidence intervals were calculated for infections when the number of deaths within 60 days in every 12 month period was greater than five. Age-sex adjusted comparisons of the case fatality rates before and after 1 March 2020 were conducted using logistic regression, then further adjusted for the comorbidities listed in table 1, with further sensitivity analyses excluding people with a concurrent or previous hospital diagnosis of covid-19. Analyses were undertaken using Stata 16/MP.

### Data completeness

NHS Digital are the custodians and suppliers of HES data, and we receive monthly updates but with a three month lag, meaning that a data cut received at the end of September 2021 will cover hospital admissions up to 30 June 2021. A comparison of different data cuts revealed that numbers of admissions in the most recent month of the penultimate data cut were, on average, around 84% of the numbers for the same month in the latest data cut (the numbers for May received at the end of August were about 84% of the numbers for May received at the end of September). This issue of completeness did not extend beyond the latest month of data for which numbers differed by no more than 1%. To account for this incompleteness in the final month, we applied an uplift to the final month’s numbers to obtain corrected numbers by applying the following calculation in relation to each infection: Obs/(C%), where Obs is the number of hospital admissions observed in the final month of the latest data cut and C% is that month’s estimated completeness based on a comparison of the latest month of the penultimate data cut and the penultimate month of the latest data cut. The same method was applied with each new data cut received. Combining all conditions, a valid value for ethnic category was present on 97.4% of records; age, sex, region, and deprivation level were complete on all records.

### Patient and public involvement

Neither patients nor the public were involved in developing the research question or in the design, management, or interpretation of this study. The primary barrier was the rapid timescale of analysis to deliver timely results.

## Results

For all infections, the demographic characteristics of patients admitted in the 12 months after 1 March 2020 were similar to those observed from 1 March 2017 to 29 February 2020 except that the age distribution of admissions for pneumonia shifted more towards infants after 1 March 2020 (supplementary table 2). The proportion who had underlying comorbidities remained largely unchanged throughout the study period.

**Fig 1:**
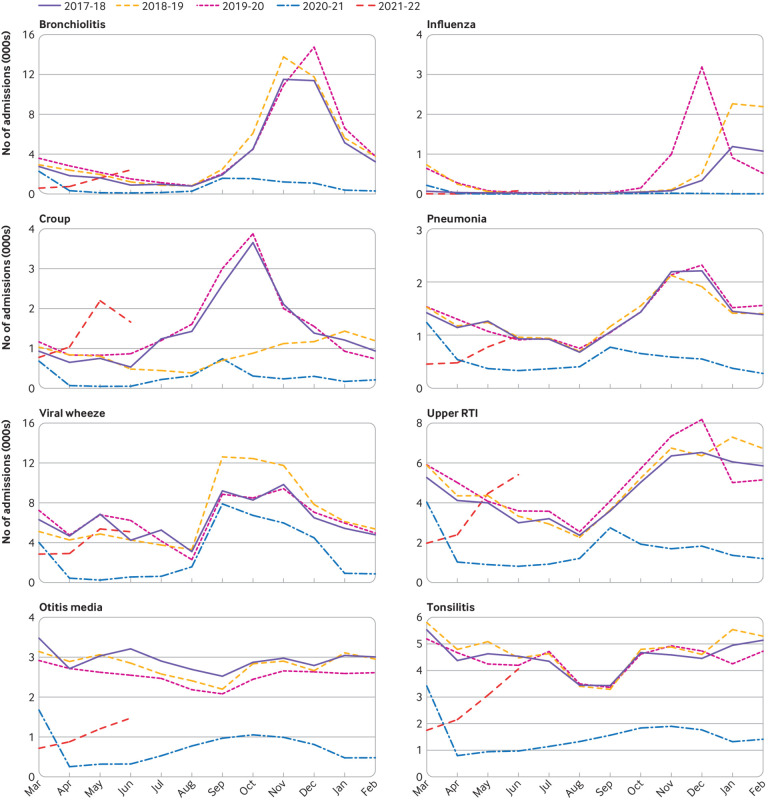
Monthly admissions for respiratory infections in England from March 2017 to June 2021. RTI=respiratory tract infection

**Fig 2:**
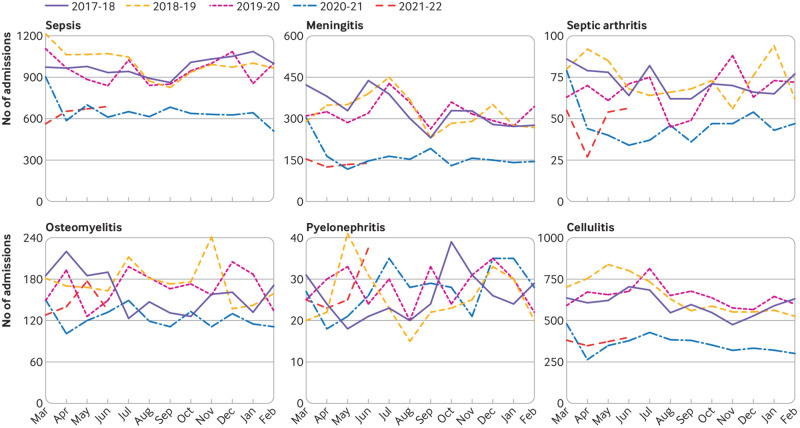
Monthly admissions for severe invasive infections in England from March 2017 to June 2021

**Fig 3:**
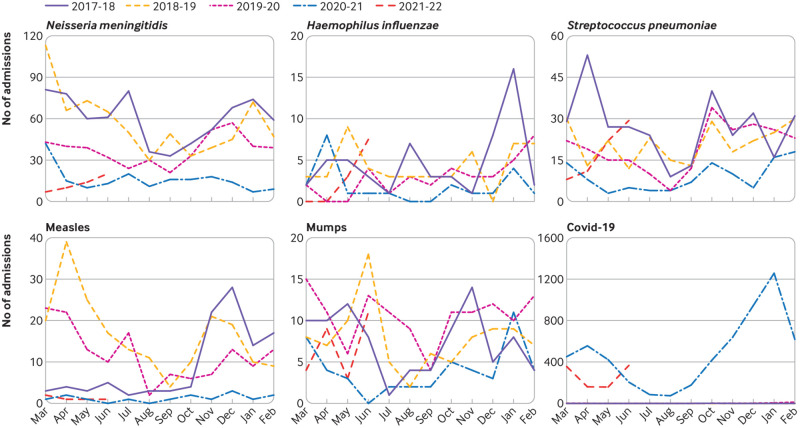
Monthly admissions for vaccine preventable infections in England from March 2017 to June 2021

**Fig 4:**
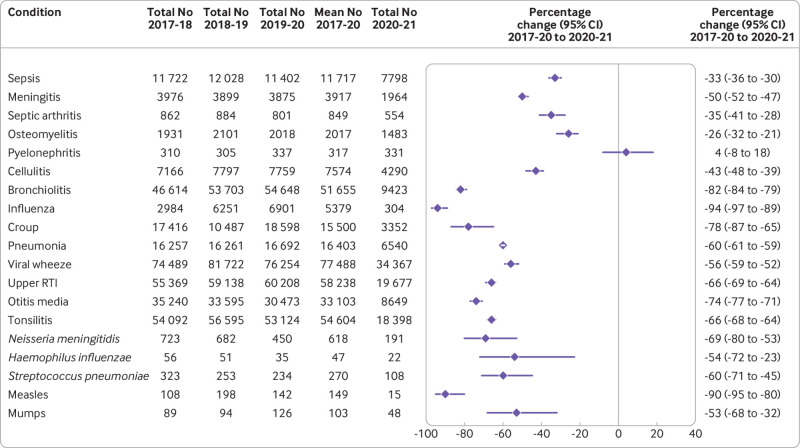
Number of hospital admissions in each 12 month period (March-February), and percentage change from March 2017-February 2020 (mean over three years) to March 2020-February 2021. RTI=respiratory tract infection

Figure 1, figure 2, and figure 3 show the number of admissions for each infection in each month from March 2017 to June 2021. In the 12 months from 1 March 2020, major reductions were found compared with the preceding 36 months in the numbers of admissions for every infection studied except pyelonephritis (fig 4). Boxplots indicated that the reductions were similar across NHS hospital trusts (supplementary fig 1).

Among the respiratory infections, the greatest percentage reduction was for influenza where the number of hospital admissions decreased by 94% (95% confidence interval 89% to 97%) from 5379 (annual mean from 1 March 2017 to 29 February 2020) to 304 in the 12 months after 1 March 2020. Additionally, for bronchiolitis admissions decreased by more than 80% (79% to 84%) from 51 655 (annual mean 2017-20) to 9423 in 2020-21. For all respiratory infections, reductions were seen in all geographical regions, index of multiple deprivation classifications, and ethnic groups (supplementary fig 2). Similar reductions in admissions for respiratory conditions occurred among children with comorbidities, including asthma, bronchopulmonary dysplasia, cystic fibrosis, extremely preterm infants, and those with haematological malignancies. Compared with the mean number of admissions in the corresponding months of 2017-19, admissions for croup were 2.8 times higher than usual in May 2021 (95% confidence interval 2.6 to 2.9) and 2.3 times higher than usual in June 2021 (1.4 to 3.8), and admissions for upper respiratory tract infections in June 2021 were 1.4 times higher than usual (1.2 to 1.6).

For five of the six severe invasive infections studied (all except pyelonephritis), substantial decreases were observed in the number of admissions. Percentage reductions ranged from 26% (95% confidence interval 21% to 32%) for osteomyelitis to 50% (47% to 52%) for meningitis; similar reductions were seen across all geographical regions, index of multiple deprivation classifications, and ethnic groups (supplementary fig 3).

Among the vaccine preventable infections (fig 4; supplementary fig 4), the greatest reduction was for measles where the number of admissions decreased by 90% (95% confidence interval 80% to 95%) from 149 (annual mean 2017-2020) to 15 in 2020-21. Admissions for *Neisseria meningitidis* decreased by 69% (53% to 80%), while admissions for *Streptococcus pneumoniae*, *Haemophilus influenzae*, and mumps more than halved. Admissions with a diagnosis of covid-19 peaked in January 2021 (fig 4).

There were six infective conditions where the number of 60 day case fatalities exceeded five in each 12 month period: sepsis, meningitis, bronchiolitis, pneumonia, viral wheeze, and upper respiratory tract infections. Alongside the decrease in admissions for these conditions, there was a decrease in the absolute number of 60 day fatalities. For pneumonia, although the absolute number of 60 day fatalities decreased (from a three year average of 193 in 2017-20 to 156 after 1 March 2020), the proportion admitted who died within 60 days increased after the onset of the pandemic (age-sex adjusted odds ratio 1.73, 95% confidence interval 1.42 to 2.11). Further adjusting for comorbidities attenuated the odds ratio (1.62, 1.35 to 1.94); additionally removing 455 patients who had a hospital recorded diagnosis of covid-19 (representing 7% of the total admissions with pneumonia in 2020-21) did not alter the effect size further (1.61, 1.34 to 1.94; table 2).

**Table 2:** Sixty day case fatalities in each 12 month period (March to February) for conditions with case fatality counts exceeding five each year

Condition	60 day case fatalities	March 2017 to February2018	March 2018 to February 2019	March 2019 to February 2020	March 2020 to February 2021		Model 1*			Model 2*	
							Adjusted odds ratio (95% CI)	P value		Adjusted odds ratio (95% CI)	P value
Sepsis	No of deaths/cases	148/11 722	163/12 028	158/11 402	110/7798		1.03 (0.84 to 1.27)	0.77		1.00 (0.81 to 1.24)	0.98
	Rate per 10 000 cases (95% CI)	126 (107 to 148)	136 (116 to 158)	139 (118 to 162)	141 (116 to 170)						
Meningitis	No of deaths/cases	29/3976	38/3899	31/3875	21/1964		1.28 (0.79 to 2.05)	0.31		1.07 (0.66 to 1.73)	0.78
	Rate per 10 000 cases (95% CI)	73 (49 to 105)	97 (69 to 134)	80 (54 to 113)	107 (66 to 163)						
Bronchiolitis	No of deaths/cases	69/46 614	55/53 703	65/54 648	16/9423		1.35 (0.81 to 2.26)	0.25		1.18 (0.71 to 1.98)	0.52
	Rate per 10 000 cases (95% CI)	15 (12 to 19)	10 (8 to 13)	12 (9 to 15)	17 (10 to 28)						
Pneumonia	No of deaths/cases	196/16 257	212/16 261	172/16 692	156/6540		1.71 (1.43 to 2.05)	P<0.001		1.62 (1.35 to 1.94)	P<0.001
	Rate per 10 000 cases (95% CI)	121 (104 to 139)	130 (114 to 149)	103 (88 to 120)	239 (203 to 278)						
Viral wheeze	No of deaths/cases	17/74 489	14/81 722	14/76 254	9/34 367		1.47 (0.71 to 3.01)	0.30		1.51 (0.73 to 3.1)	0.26
	Rate per 10 000 cases (95% CI)	2 (1 to 4)	2 (1 to 3)	2 (1 to 3)	3 (1 to 5)						
Upper RTI	No of deaths/cases	40/55 369	42/59 138	26/60 208	15/19 677		1.22 (0.71 to 2.09)	0.48		1.19 (0.69 to 2.04)	0.53
	Rate per 10 000 cases (95% CI)	7 (5 to 10)	7 (5 to 10)	4 (3 to 6)	8 (4 to 13)						

RTI=respiratory tract infection.

* Model 1: adjusted for age and sex; model 2: adjusted for age, sex, and comorbidities in table 1. All odds ratios compare 60 day case fatality from March 2020 to February 2021 with reference period from March 2017 to February 2020.

## Discussion

### Principal findings

Since the onset of the covid-19 pandemic, there have been major reductions in hospital admissions for respiratory, severe invasive, and vaccine preventable infections in children in England. These reductions were seen in all demographic subgroups, including in children with pre-existing comorbidities who are more likely to be admitted to hospital with severe disease, require prolonged intensive care support, and be at greatest risk from dying of infection.

The findings indicate the extent to which measures related to the control of covid-19 can also impact on the transmission of other specific infectious diseases in childhood. Before the pandemic, bronchiolitis accounted for 12% of all admissions to paediatric intensive care units and had a case fatality rate of 1.75% in children aged <12 months and 4.4% in children aged ≥12 months.^22^ Exacerbation of asthma caused by acute respiratory infections was also one of the commonest reasons for hospital admission in children. In this study, after 1 March 2020, we report substantial reductions in admissions for bronchiolitis (80%), viral wheeze (56%), and predominantly bacterial causes of childhood respiratory infection: 60% reduction in the overall number of children admitted with pneumonia, 74% reduction for otitis media, and 66% reduction for tonsillitis. Children with haematological malignancies and cystic fibrosis who are most susceptible to major bacterial infections also had lower incidence of admission for respiratory infections compared with previous years (supplementary fig 2). Children with respiratory infections account for high healthcare costs, substantial morbidity, and in rare instances, mortality.

### Contributing causes and implications for the future

While the pattern of hospital recorded covid-19 is probably almost entirely driven by universal testing for the infection at the point of admission for any medical or surgical condition, the dramatic reduction in transmission rates of non-covid-19 infectious pathogens among children is probably because of changes in patterns of social contact (social distancing, school closures, and minimal recreational activities) and shielding measures (at risk groups). Vastly restricted domestic and international travel (reduced air pollution) might have also contributed to the reduction in childhood respiratory infections.^16^ ^23^ However, these measures are generally unsustainable outside of the pandemic because of their inherent negative financial and societal impact.

The immediate and substantial reduction in respiratory and severe invasive childhood infections is also probably caused by the widespread introduction of non-pharmacological interventions. A range of behavioural measures, including face masks, better and regular hand hygiene, improved respiratory etiquette, and environmental cleaning have been adopted across homes, schools, and workplaces in England, which helped in reducing transmission of SARS-CoV-2.^24^ Our data indicate that these interventions might have also minimised childhood transmission of non-covid-19 infections. In particular, this study has highlighted that children with severe underlying comorbidities, including extremely preterm infants, those with congenital cardiac disease, and those with asthma have been protected from severe and potentially life threatening infection.

School closures might have contributed to some of the reductions seen in this study, but it was not possible to disentangle the effects of school closures and other policies around physical distancing. School closures during influenza pandemics have been shown to reduce epidemic peaks, but conflicting evidence exists about optimal timing and duration. The success of school closures is largely dependent on the characteristics of the circulating influenza serotype and when transmission is higher in children than adults.^24^ ^25^ National surveillance data in 11 966 children from 131 schools in England showed extremely low rates of SARS-CoV-2 infection and transmission in children aged up to 11 years at the point of school closure and also at reopening.^26^ During the covid-19 pandemic, good hygiene practices have been implemented (hand sanitisers in classrooms, regular hand washing, education on respiratory etiquette) along with measures to minimise spread of infection (avoiding overcrowding and good ventilation in classrooms). Furthermore, children with respiratory symptoms were excluded until confirmed to have a negative covid-19 test. Ensuring children with symptoms are off school, and encouraging employers to support parents to be at home with their unwell child, could help reduce the burden of childhood infections transmitted at school. However, a systematic review that included 72 observational studies assessed the impact of school closures on the mental health of children during covid-19 and showed increased anxiety, depressive and emotional behaviours.^27^ Preliminary observational data from France suggest there had been no increase in the rates of common respiratory infections among children immediately after the reopening of schools after national lockdown measures were lifted^15^; this could be because of societal behaviours persisting immediately after schools reopened and unwell children being kept home. Further analysis is required to assess which measures might be maintained in future to reduce transmission of infections more broadly while avoiding the negative impact of closing schools. Ongoing clinical and microbiological surveillance is required to evaluate any epidemiological changes (eg, substantial increase in rates, delayed peaks, disease in older infants) in respiratory infections that might have occurred because of a lack of population based immunity during the pandemic. The unusually high rates of croup and upper respiratory tract infections in the last months of this study (in May and June 2021) further underline this need.

The reduction in musculoskeletal infections could be because of decreased exposure to *Staphylococcus aureus* and *Kingella kingae*, which are the commonest causative pathogens. The larger reductions seen in hospital admission due to cellulitis, compared with septic arthritis or osteomyelitis, might be because these children were treated with oral antibiotics and could be more easily managed at home compared with those with bone and joint infection. The epidemiology of bone and joint infections in children is poorly understood because of a lack of clinical or microbiological surveillance. These data could encourage future studies to evaluate the cause (eg, contribution of preceding respiratory infections, trauma, or underlying comorbidities) to better inform management strategies.

Our data also show important reductions in respiratory infections among children with pre-existing lung disease. To obtain a holistic overview of protective mechanisms that have reduced hospital admissions for respiratory infections in these patients, there is a need for more quantitative data (changes in patterns to elective admissions, exposure to respiratory pathogens, colonisation with resistant pathogens) and qualitative data (from children and their caregivers).

The absence of any reduction in admissions for pyelonephritis might be because non-pharmacological interventions and social restrictions have no impact on this condition; the slight percentage increase (4%, 95% confidence interval −8% to 18%), though not statistically significant, could be caused by delays in presentation for children who had uncomplicated urinary tract infections that might have progressed to ascending infection by the time of hospital attendance.

Reductions in bacterial pathogens during the covid-19 pandemic, which have been reported in laboratory confirmed data from 26 countries worldwide,^28^ might be caused by fewer social interactions limiting the transmission of vaccine preventable infections.^29^ ^30^ However, these reductions might also be partly caused by reduced circulating respiratory viral infections (in particular, respiratory syncytial virus and influenza), which can increase susceptibility to pneumococcal and meningococcal disease. In regions with interrupted vaccination programmes, active enhanced surveillance mechanisms and catch up vaccination programmes should be prioritised to identify any resurgence in vaccine preventable infections and ensure herd immunity is maintained.

Reduced hospital attendance could be partly due to reluctance to attend the emergency department during a pandemic and potentially being exposed to SARS-CoV-2. Reassuringly, this study indicates that the pandemic has not had an adverse effect on absolute numbers of fatalities because deaths within 60 days have also reduced alongside hospital admissions (investigation of death rates from infections among children who did not have a related hospital admission was outside the scope of this study). However, for pneumonia, although the absolute number of 60 day fatalities decreased, the proportion admitted who died within 60 days increased. This increase could be because hospital admissions for pneumonia after 1 March 2020 were generally at the more severe end of the spectrum if people with less severe disease were being more frequently managed outside hospital or because of delays in seeking or difficulty in accessing appropriate healthcare during the pandemic. At the Royal Children’s Hospital in Melbourne, the Hospital in The Home service observed that a dedicated outpatient team can safely manage children with meningitis, severe cellulitis, and complex urinary tract infections in their home.^31^ ^32^ ^33^ This service has been shown to deliver cost savings to the healthcare provider and improves quality of life for the child and caregiver. Future work should include caregiver perceptions in seeking healthcare during the pandemic to minimise anxiety, ensure clear public health messaging that encourages caregivers to attend emergency departments when necessary, and develop outpatient services to manage children with infection in the community if appropriate.

### Strengths and limitations

The strengths of this study include the national coverage of data available because they capture all relevant hospital admissions for the entire child population of England over several years; determination of underlying comorbidities based on patients’ entire previous NHS hospital admission history; and the inclusion of a range of different infections providing the means to compare them. This study has some limitations. The reliability of HES data is dependent on information collected from clinical notes and clinical coding practice, which is important when comparing data from one year to the next because the quality might vary. Beyond the clinical diagnoses (ICD-10 codes), no other clinical or microbiological data were available. Therefore, disease severity (such as admission to paediatric intensive care unit) could not be evaluated and ICD codes could not be corroborated with laboratory results. The HES data represent hospital admissions for infection and do not necessarily provide a complete picture of the true rates of actual infection in the population as they do not cover patients who did not attend an NHS hospital (eg, owing to patient or caregiver reluctance or possible shortage of healthcare provision) or those who only attended the emergency department or outpatient settings (that is, they did not require inpatient admission). For comorbidity data, although we were able to look back historically at each patient’s entire NHS hospital admission history, comorbidity profiles might be incomplete because the data did not cover emergency department or outpatient settings (only inpatient admissions and day cases), primary care, or non-NHS care.

### Comparison with other studies

This large epidemiological study evaluates the impact of covid-19 on hospital admission rates for childhood infections. Similar findings have been reported in regional studies in Australia, Denmark, France, and the Netherlands, with reported reductions of more than 50% in paediatric attendances with common infectious diseases.^34^ ^35^ ^36^ However, it would be valuable to establish similar continuous analyses in other locations where centralised electronic health record data are available (eg, China, Taiwan, or Sweden, or in health maintenance organisations in the United States) to monitor such trends until the challenges associated with the pandemic diminish.

### Conclusions

This study has shown dramatic overall reductions in hospital admissions for respiratory, severe invasive, and vaccine preventable infections in children during the covid-19 pandemic in England. Children with potentially life threatening comorbidities were also substantially protected. Further evaluation of non-pharmacological interventions that could be sustained beyond the pandemic is required to inform policy makers about potential strategies, especially during winter months, to minimise the burden on health systems and protect vulnerable children. Continued monitoring of hospital admissions for these infections is required as social restrictions evolve.

**What is already known on this topic**

Childhood immunisation programmes in high and low income countries have been disrupted since the onset of the covid-19 pandemic owing to barriers in accessing or administering vaccines
Data from laboratory surveillance studies indicate worldwide reductions in some childhood infections since the onset of the pandemic
Reports have shown delayed presentations to paediatric emergency departments in the UK and in other countries, but the impact of such delays on patient outcomes is unclear

**What this study adds**

In the 12 months after the onset of the covid-19 pandemic, large and sustained reductions were found in rates of hospital admissions for a wide range of severe, respiratory, and vaccine preventable childhood infections in England
Absolute numbers of deaths within 60 days of hospital admission for sepsis, meningitis, bronchiolitis, pneumonia, viral wheeze, and upper respiratory tract infections also decreased
More recent data indicate that croup and upper respiratory tract infections increased to higher levels than usual after May 2021

## Data Availability

The dataset used in this study can be obtained by successful application to NHS Digital.

## Web extra

Extra material supplied by authors

Web appendix: Supplementary materials

## Notes

Contributors: SK and RG contributed equally as joint first authors. All authors conceived the study question. SK and RG wrote the first draft of the manuscript. SK conducted the literature review. RG conducted the analyses. All authors contributed to subsequent drafts and interpretation of the findings. SK and RG are study guarantors. The corresponding author attests that all listed authors meet authorship criteria and that no others meeting the criteria have been omitted.

Dissemination to participants and related patient and public communities: The project team will continue to update the analyses based on these data sources and publish them (alongside any developments to the methods used) at https://www.ctsu.ox.ac.uk/research/indirect-effects-of-the-covid-19-pandemic-on-childhood-infection-in-england. This study will also be disseminated by our institutions’ social media channels.

The lead authors (the manuscript’s guarantors) affirm that the manuscript is an honest, accurate, and transparent account of the study being reported; that no important aspects of the study have been omitted; and that any discrepancies from the study as planned (and, if relevant, registered) have been explained.

Provenance and peer review: Not commissioned; externally peer reviewed.
