# Supplementary material for: Indirect effects of the covid-19 pandemic on childhood infection in England: population based observational study
Source: BMJ. 2022 Jan 12;376:e067519. doi: 10.1136/bmj-2021-067519 (PMC8753487; doi:10.1136/bmj-2021-067519)
Supplement: Supplementary file 1 — Web appendix: Supplementary materials [file kads067519.ww.pdf]

Supplementary Table 1: Estimated population size of children aged 0-14 in England 2017-2020 (estimates for 2021 were not yet available at the time of publication)

Year	Aged <1	Aged 1-4	Aged 5-9	Aged 10-14	Total
**2017**	653,467	2,731,458	3,497,402	3,166,038	10,048,365
**2018**	637,834	2,708,893	3,523,866	3,274,119	10,144,712
**2019**	618,858	2,680,779	3,538,206	3,354,246	10,192,089
**2020**	601,913	2,637,534	3,539,458	3,435,579	10,214,484

Source: Office for National Statistics

https://www.ons.gov.uk/file?uri=/peoplepopulationandcommunity/populationandmigration/populationestimates/datasets/populationestimatesforukenglandandwalesscotlandandnorthernireland/mid2001tomid2020detailedtimeseries/regionalpopestimatesenglandandwales19712020.xlsx

https://www.ons.gov.uk/file?uri=%2Fpeoplepopulationandcommunity%2Fpopulationandmigration%2Fpopulationestimates%2Fdatasets%2Fpopulationestimatesforukenglandandwalesscotlandandnorthernireland%2Fmid2020/ukpopestimatesmid2020on2021geography.xls

Supplementary table 2a: Demographic characteristics of individuals admitted with each respiratory infection in 2017-20 and in 2020-21

	Bronchiolitis		Influenza		Croup		Pneumonia		Viral wheeze		Upper RTI		Otitis media		Tonsilitis	
	2017-20	2020-21	2017-20	2020-21	2017-20	2020-21	2017-20	2020-21	2017-20	2020-21	2017-20	2020-21	2017-20	2020-21	2017-20	2020-21
	N (% of total)	N (% of total)	N (% of total)	N (% of total)	N (% of total)	N (% of total)	N (% of total)	N (% of total)	N (% of total)	N (% of total)	N (% of total)	N (% of total)	N (% of total)	N (% of total)	N (% of total)	N (% of total)
**Total number:**	51,655	9,423	5,379	304	15,500	3,352	16,403	6,540	77,488	34,367	58,238	19,677	33,103	8,649	54,604	18,398
**Sex**																
Male	31415 (60.8)	6270 (66.5)	2955 (54.9)	165 (54.3)	10645 (68.7)	2500 (74.6)	8885 (54.2)	3720 (56.9)	50125 (64.7)	23020 (67)	32240 (55.4)	11045 (56.1)	19110 (57.7)	5090 (58.9)	29060 (53.2)	9920 (53.9)
Female	20240 (39.2)	3155 (33.5)	2425 (45.1)	140 (46.1)	4855 (31.3)	850 (25.4)	7520 (45.8)	2820 (43.1)	27365 (35.3)	11350 (33)	26000 (44.6)	8635 (43.9)	13995 (42.3)	3560 (41.2)	25545 (46.8)	8475 (46.1)
**Age group**																
<1	46125 (89.3)	7780 (82.6)	1070 (19.9)	55 (18.1)	3805 (24.5)	845 (25.2)	4300 (26.2)	2530 (38.7)	6740 (8.7)	1730 (5)	20090 (34.5)	7460 (37.9)	2060 (6.2)	810 (9.4)	5410 (9.9)	2100 (11.4)
1-4	5425 (10.5)	1610 (17.1)	2575 (47.9)	150 (49.3)	10160 (65.5)	2055 (61.3)	7895 (48.1)	2055 (31.4)	61795 (79.7)	26835 (78.1)	30510 (52.4)	9900 (50.3)	16495 (49.8)	4105 (47.5)	29775 (54.5)	10765 (58.5)
5-9	80 (0.2)	20 (0.2)	1030 (19.1)	60 (19.7)	1335 (8.6)	390 (11.6)	2795 (17)	1125 (17.2)	7785 (10)	5060 (14.7)	5615 (9.6)	1600 (8.1)	11685 (35.3)	2715 (31.4)	13500 (24.7)	3795 (20.6)
10-14	30 (0.1)	15 (0.2)	705 (13.1)	45 (14.8)	200 (1.3)	60 (1.8)	1415 (8.6)	835 (12.8)	1170 (1.5)	740 (2.2)	2020 (3.5)	715 (3.6)	2865 (8.7)	1025 (11.9)	5920 (10.8)	1740 (9.5)
**Region of residence**																
North East	2865 (5.5)	400 (4.2)	220 (4.1)	5 (1.6)	955 (6.2)	175 (5.2)	625 (3.8)	185 (2.8)	3720 (4.8)	1655 (4.8)	3750 (6.4)	1055 (5.4)	1950 (5.9)	510 (5.9)	2870 (5.3)	930 (5.1)
North West	9765 (18.9)	1790 (19)	1325 (24.6)	65 (21.4)	3065 (19.8)	660 (19.7)	1700 (10.4)	675 (10.3)	13050 (16.8)	5755 (16.7)	12875 (22.1)	4400 (22.4)	5645 (17.1)	1540 (17.8)	11440 (21)	3715 (20.2)
Yorkshire and Humber	5095 (9.9)	960 (10.2)	480 (8.9)	30 (9.9)	1425 (9.2)	285 (8.5)	1725 (10.5)	685 (10.5)	6315 (8.1)	2890 (8.4)	5230 (9)	1730 (8.8)	3625 (11)	860 (9.9)	5850 (10.7)	1820 (9.9)
East Midlands	4030 (7.8)	690 (7.3)	365 (6.8)	20 (6.6)	1200 (7.7)	210 (6.3)	1215 (7.4)	440 (6.7)	6410 (8.3)	2640 (7.7)	4020 (6.9)	1195 (6.1)	2525 (7.6)	555 (6.4)	3995 (7.3)	1170 (6.4)
West Midlands	5735 (11.1)	985 (10.5)	500 (9.3)	25 (8.2)	1825 (11.8)	330 (9.8)	1580 (9.6)	670 (10.2)	8680 (11.2)	3855 (11.2)	7205 (12.4)	2020 (10.3)	3390 (10.2)	870 (10.1)	6300 (11.5)	1880 (10.2)
East of England	5480 (10.6)	990 (10.5)	425 (7.9)	25 (8.2)	1400 (9)	390 (11.6)	1540 (9.4)	585 (8.9)	6695 (8.6)	3320 (9.7)	5485 (9.4)	2505 (12.7)	4070 (12.3)	1035 (12)	6105 (11.2)	2515 (13.7)
London	6210 (12)	1175 (12.5)	895 (16.6)	70 (23)	1755 (11.3)	350 (10.4)	3475 (21.2)	1410 (21.6)	12765 (16.5)	5215 (15.2)	5990 (10.3)	1555 (7.9)	3675 (11.1)	905 (10.5)	6000 (11)	1720 (9.3)
South East	7560 (14.6)	1480 (15.7)	605 (11.2)	40 (13.2)	2365 (15.3)	485 (14.5)	2740 (16.7)	1225 (18.7)	12365 (16)	5440 (15.8)	7425 (12.7)	2510 (12.8)	5425 (16.4)	1470 (17)	8005 (14.7)	2885 (15.7)
South West	4915 (9.5)	955 (10.1)	560 (10.4)	25 (8.2)	1510 (9.7)	465 (13.9)	1800 (11)	660 (10.1)	7490 (9.7)	3605 (10.5)	6265 (10.8)	2705 (13.7)	2795 (8.4)	905 (10.5)	4045 (7.4)	1765 (9.6)
**Deprivation quintile**																
1 (Most deprived)	15890 (30.8)	2815 (29.9)	1785 (33.2)	100 (32.9)	3950 (25.5)	815 (24.3)	4385 (26.7)	1850 (28.3)	22780 (29.4)	10265 (29.9)	17295 (29.7)	5405 (27.5)	8320 (25.1)	2125 (24.6)	15930 (29.2)	4695 (25.5)
2	11275 (21.8)	2045 (21.7)	1180 (21.9)	60 (19.7)	3180 (20.5)	660 (19.7)	3670 (22.4)	1460 (22.3)	16980 (21.9)	7305 (21.3)	12775 (21.9)	4035 (20.5)	6865 (20.7)	1840 (21.3)	11855 (21.7)	3990 (21.7)
3	9200 (17.8)	1680 (17.8)	905 (16.8)	50 (16.4)	2915 (18.8)	660 (19.7)	3105 (18.9)	1125 (17.2)	13935 (18)	6390 (18.6)	10565 (18.1)	3750 (19.1)	6195 (18.7)	1580 (18.3)	10005 (18.3)	3465 (18.8)
4	8065 (15.6)	1530 (16.2)	785 (14.6)	45 (14.8)	2800 (18.1)	640 (19.1)	2625 (16)	1115 (17)	12110 (15.6)	5205 (15.1)	9350 (16.1)	3345 (17)	5905 (17.8)	1655 (19.1)	8865 (16.2)	3195 (17.4)
5 (Least deprived)	7230 (14)	1355 (14.4)	725 (13.5)	50 (16.4)	2655 (17.1)	580 (17.3)	2610 (15.9)	990 (15.1)	11685 (15.1)	5205 (15.1)	8255 (14.2)	3145 (16)	5820 (17.6)	1445 (16.7)	7950 (14.6)	3055 (16.6)
**Ethnic category**																
White	39525 (76.5)	7100 (75.3)	3570 (66.4)	185 (60.9)	12385 (79.9)	2715 (81)	11230 (68.5)	4345 (66.4)	52385 (67.6)	22180 (64.5)	43005 (73.8)	14775 (75.1)	26620 (80.4)	6895 (79.7)	41480 (76)	14345 (78)
Mixed	2475 (4.8)	425 (4.5)	250 (4.6)	20 (6.6)	645 (4.2)	155 (4.6)	775 (4.7)	350 (5.4)	4525 (5.8)	2205 (6.4)	2700 (4.6)	1005 (5.1)	1170 (3.5)	330 (3.8)	2330 (4.3)	890 (4.8)
Asian	5240 (10.1)	970 (10.3)	895 (16.6)	55 (18.1)	1265 (8.2)	225 (6.7)	2275 (13.9)	945 (14.4)	12250 (15.8)	5805 (16.9)	7070 (12.1)	2015 (10.2)	2950 (8.9)	795 (9.2)	5880 (10.8)	1490 (8.1)
Black	1760 (3.4)	345 (3.7)	325 (6)	30 (9.9)	385 (2.5)	65 (1.9)	1065 (6.5)	450 (6.9)	3885 (5)	1830 (5.3)	2125 (3.6)	620 (3.2)	810 (2.4)	200 (2.3)	1730 (3.2)	520 (2.8)
Other	1200 (2.3)	285 (3)	210 (3.9)	15 (4.9)	410 (2.6)	85 (2.5)	655 (4)	255 (3.9)	2785 (3.6)	1340 (3.9)	1665 (2.9)	545 (2.8)	795 (2.4)	215 (2.5)	1690 (3.1)	560 (3)
**Comorbidity**																
No known comorbidity	47055 (91.1)	8255 (87.6)	4335 (80.6)	240 (78.9)	13915 (89.8)	2890 (86.2)	12545 (76.5)	4625 (70.7)	65225 (84.2)	29120 (84.7)	51435 (88.3)	17360 (88.2)	29075 (87.8)	7460 (86.3)	49435 (90.5)	16885 (91.8)
Asthma	255 (0.5)	80 (0.8)	430 (8)	25 (8.2)	740 (4.8)	220 (6.6)	1640 (10)	625 (9.6)	9395 (12.1)	4185 (12.2)	2825 (4.9)	885 (4.5)	2520 (7.6)	635 (7.3)	3580 (6.6)	950 (5.2)
Bronchopulmonary dysplasia	1205 (2.3)	340 (3.6)	110 (2)	5 (1.6)	165 (1.1)	50 (1.5)	510 (3.1)	260 (4)	955 (1.2)	295 (0.9)	745 (1.3)	250 (1.3)	240 (0.7)	80 (0.9)	280 (0.5)	70 (0.4)
Extreme prematurity <28 weeks	1440 (2.8)	365 (3.9)	95 (1.8)	5 (1.6)	200 (1.3)	50 (1.5)	485 (3)	270 (4.1)	995 (1.3)	325 (0.9)	750 (1.3)	250 (1.3)	190 (0.6)	75 (0.9)	285 (0.5)	95 (0.5)
Cystic fibrosis	55 (0.1)	15 (0.2)	35 (0.7)	5 (1.6)	20 (0.1)	5 (0.1)	80 (0.5)	35 (0.5)	70 (0.1)	35 (0.1)	150 (0.3)	45 (0.2)	25 (0.1)	20 (0.2)	70 (0.1)	25 (0.1)
Congenital cardiac disease	3090 (6)	790 (8.4)	355 (6.6)	30 (9.9)	645 (4.2)	190 (5.7)	1595 (9.7)	865 (13.2)	2090 (2.7)	810 (2.4)	2730 (4.7)	1050 (5.3)	1170 (3.5)	430 (5)	1165 (2.1)	425 (2.3)
Bronchiectasis	40 (0.1)	15 (0.2)	35 (0.7)	5 (1.6)	10 (0.1)	0 (0)	120 (0.7)	80 (1.2)	145 (0.2)	55 (0.2)	80 (0.1)	40 (0.2)	50 (0.2)	25 (0.3)	30 (0.1)	15 (0.1)
Immunodeficiencies	25 (0)	15 (0.2)	25 (0.5)	0 (0)	25 (0.2)	5 (0.1)	70 (0.4)	60 (0.9)	95 (0.1)	55 (0.2)	115 (0.2)	40 (0.2)	80 (0.2)	30 (0.3)	80 (0.1)	35 (0.2)
Acute lymphoblastic leukaemia	15 (0)	0 (0)	170 (3.2)	10 (3.3)	40 (0.3)	10 (0.3)	170 (1)	195 (3)	45 (0.1)	25 (0.1)	600 (1)	195 (1)	195 (0.6)	80 (0.9)	120 (0.2)	45 (0.2)
Acute myeloid leukaemia	5 (0)	0 (0)	15 (0.3)	0 (0)	5 (0)	0 (0)	55 (0.3)	30 (0.5)	10 (0)	0 (0)	50 (0.1)	20 (0.1)	20 (0.1)	10 (0.1)	15 (0)	5 (0)

Subgroup numbers are rounded to the nearest 5, in accordance with NHS Digital reporting guidelines, to preclude deductive identification of individual patients where numbers are small

Supplementary table 2b: Demographic characteristics of individuals admitted with each severe invasive infection in 2017-20 and in 2020-21

	Sepsis		Meningitis		Septic arthritis		Osteomyelitis		Pyelonephritis		Cellulitis	
	2017-20	2020-21	2017-20	2020-21	2017-20	2020-21	2017-20	2020-21	2017-20	2020-21	2017-20	2020-21
	N (% of total)	N (% of total)	N (% of total)	N (% of total)	N (% of total)	N (% of total)	N (% of total)	N (% of total)	N (% of total)	N (% of total)	N (% of total)	N (% of total)
**Total number:**	11,717	7,798	3,917	1,964	849	554	2,017	1,483	317	331	7,574	4,290
**Sex**												
Male	6485 (55.3)	4470 (57.3)	2260 (57.7)	1040 (53)	475 (55.9)	310 (56)	1075 (53.3)	760 (51.2)	65 (20.5)	80 (24.2)	4110 (54.3)	2310 (53.8)
Female	5230 (44.6)	3330 (42.7)	1655 (42.3)	920 (46.8)	370 (43.6)	245 (44.2)	945 (46.9)	725 (48.9)	250 (78.9)	250 (75.5)	3465 (45.7)	1980 (46.2)
**Age group**												
<1	6000 (51.2)	4070 (52.2)	2435 (62.2)	1150 (58.6)	130 (15.3)	95 (17.1)	130 (6.4)	65 (4.4)	40 (12.6)	60 (18.1)	1165 (15.4)	775 (18.1)
1-4	3285 (28)	1990 (25.5)	565 (14.4)	270 (13.7)	355 (41.8)	195 (35.2)	430 (21.3)	275 (18.5)	65 (20.5)	80 (24.2)	2775 (36.6)	1370 (31.9)
5-9	1460 (12.5)	945 (12.1)	505 (12.9)	275 (14)	180 (21.2)	120 (21.7)	545 (27)	385 (26)	105 (33.1)	105 (31.7)	1935 (25.5)	1120 (26.1)
10-14	975 (8.3)	795 (10.2)	415 (10.6)	275 (14)	185 (21.8)	140 (25.3)	915 (45.4)	760 (51.2)	105 (33.1)	85 (25.7)	1700 (22.4)	1025 (23.9)
**Region of residence**												
North East	315 (2.7)	195 (2.5)	175 (4.5)	75 (3.8)	30 (3.5)	25 (4.5)	75 (3.7)	70 (4.7)	10 (3.2)	10 (3)	390 (5.1)	220 (5.1)
North West	1765 (15.1)	1225 (15.7)	495 (12.6)	245 (12.5)	105 (12.4)	80 (14.4)	280 (13.9)	190 (12.8)	35 (11)	55 (16.6)	1110 (14.7)	705 (16.4)
Yorkshire and Humber	1295 (11.1)	880 (11.3)	475 (12.1)	235 (12)	85 (10)	55 (9.9)	160 (7.9)	105 (7.1)	45 (14.2)	30 (9.1)	755 (10)	425 (9.9)
East Midlands	995 (8.5)	605 (7.8)	330 (8.4)	205 (10.4)	55 (6.5)	30 (5.4)	85 (4.2)	90 (6.1)	15 (4.7)	15 (4.5)	425 (5.6)	205 (4.8)
West Midlands	1220 (10.4)	845 (10.8)	450 (11.5)	210 (10.7)	75 (8.8)	60 (10.8)	155 (7.7)	100 (6.7)	20 (6.3)	25 (7.6)	760 (10)	425 (9.9)
East of England	1235 (10.5)	800 (10.3)	390 (10)	180 (9.2)	75 (8.8)	45 (8.1)	185 (9.2)	110 (7.4)	35 (11)	35 (10.6)	770 (10.2)	445 (10.4)
London	2265 (19.3)	1520 (19.5)	640 (16.3)	375 (19.1)	175 (20.6)	100 (18.1)	555 (27.5)	300 (20.2)	80 (25.2)	85 (25.7)	1425 (18.8)	735 (17.1)
South East	1870 (16)	1240 (15.9)	635 (16.2)	285 (14.5)	170 (20)	100 (18.1)	335 (16.6)	280 (18.9)	55 (17.4)	60 (18.1)	1355 (17.9)	740 (17.2)
South West	755 (6.4)	485 (6.2)	335 (8.6)	160 (8.1)	80 (9.4)	60 (10.8)	190 (9.4)	235 (15.8)	20 (6.3)	15 (4.5)	585 (7.7)	395 (9.2)
**Deprivation quintile**												
1 (Most deprived)	3325 (28.4)	2205 (28.3)	1045 (26.7)	590 (30)	200 (23.6)	120 (21.7)	565 (28)	390 (26.3)	90 (28.4)	105 (31.7)	2125 (28.1)	1215 (28.3)
2	2715 (23.2)	1820 (23.3)	815 (20.8)	440 (22.4)	185 (21.8)	130 (23.5)	465 (23.1)	300 (20.2)	70 (22.1)	50 (15.1)	1615 (21.3)	860 (20)
3	2110 (18)	1400 (18)	695 (17.7)	310 (15.8)	165 (19.4)	110 (19.9)	350 (17.4)	275 (18.5)	65 (20.5)	55 (16.6)	1345 (17.8)	770 (17.9)
4	1785 (15.2)	1215 (15.6)	690 (17.6)	285 (14.5)	150 (17.7)	100 (18.1)	355 (17.6)	255 (17.2)	40 (12.6)	60 (18.1)	1235 (16.3)	725 (16.9)
5 (Least deprived)	1780 (15.2)	1155 (14.8)	675 (17.2)	340 (17.3)	150 (17.7)	95 (17.1)	280 (13.9)	260 (17.5)	55 (17.4)	65 (19.6)	1255 (16.6)	725 (16.9)
**Ethnic category**												
White	8500 (72.5)	5590 (71.7)	2925 (74.7)	1375 (70)	620 (73)	400 (72.2)	1395 (69.2)	1080 (72.8)	230 (72.6)	245 (74)	5480 (72.4)	3185 (74.2)
Mixed	575 (4.9)	375 (4.8)	205 (5.2)	95 (4.8)	40 (4.7)	30 (5.4)	85 (4.2)	70 (4.7)	15 (4.7)	15 (4.5)	335 (4.4)	190 (4.4)
Asian	1460 (12.5)	975 (12.5)	390 (10)	245 (12.5)	85 (10)	75 (13.5)	215 (10.7)	130 (8.8)	40 (12.6)	40 (12.1)	870 (11.5)	435 (10.1)
Black	515 (4.4)	370 (4.7)	165 (4.2)	110 (5.6)	50 (5.9)	25 (4.5)	205 (10.2)	130 (8.8)	10 (3.2)	10 (3)	350 (4.6)	165 (3.8)
Other	430 (3.7)	295 (3.8)	135 (3.4)	100 (5.1)	40 (4.7)	15 (2.7)	95 (4.7)	50 (3.4)	20 (6.3)	10 (3)	335 (4.4)	180 (4.2)
**Comorbidity**												
No known comorbidity	9465 (80.8)	6145 (78.8)	3560 (90.9)	1665 (84.8)	770 (90.7)	505 (91.2)	1780 (88.2)	1310 (88.3)	285 (89.9)	305 (92.1)	6680 (88.2)	3695 (86.1)
Asthma	420 (3.6)	215 (2.8)	130 (3.3)	85 (4.3)	50 (5.9)	30 (5.4)	170 (8.4)	100 (6.7)	20 (6.3)	15 (4.5)	535 (7.1)	315 (7.3)
Bronchopulmonary dysplasia	240 (2)	195 (2.5)	55 (1.4)	45 (2.3)	5 (0.6)	0 (0)	10 (0.5)	5 (0.3)	0 (0)	0 (0)	40 (0.5)	40 (0.9)
Extreme prematurity <28 weeks	245 (2.1)	210 (2.7)	75 (1.9)	80 (4.1)	0 (0)	0 (0)	10 (0.5)	5 (0.3)	0 (0)	5 (1.5)	45 (0.6)	40 (0.9)
Cystic fibrosis	20 (0.2)	10 (0.1)	0 (0)	0 (0)	0 (0)	0 (0)	0 (0)	0 (0)	0 (0)	0 (0)	10 (0.1)	5 (0.1)
Congenital cardiac disease	880 (7.5)	655 (8.4)	150 (3.8)	130 (6.6)	20 (2.4)	15 (2.7)	50 (2.5)	30 (2)	5 (1.6)	10 (3)	220 (2.9)	175 (4.1)
Bronchiectasis	35 (0.3)	25 (0.3)	0 (0)	0 (0)	0 (0)	0 (0)	5 (0.2)	0 (0)	0 (0)	0 (0)	5 (0.1)	5 (0.1)
Immunodeficiencies	65 (0.6)	50 (0.6)	10 (0.3)	5 (0.3)	0 (0)	5 (0.9)	10 (0.5)	0 (0)	0 (0)	0 (0)	15 (0.2)	10 (0.2)
Acute lymphoblastic leukaemia	760 (6.5)	605 (7.8)	10 (0.3)	15 (0.8)	5 (0.6)	5 (0.9)	15 (0.7)	35 (2.4)	0 (0)	0 (0)	110 (1.5)	90 (2.1)
Acute myeloid leukaemia	125 (1.1)	90 (1.2)	5 (0.1)	0 (0)	0 (0)	0 (0)	5 (0.2)	5 (0.3)	0 (0)	0 (0)	10 (0.1)	15 (0.3)

Subgroup numbers are rounded to the nearest 5, in accordance with NHS Digital reporting guidelines, to preclude deductive identification of individual patients where numbers are small

Supplementary table 2c: Demographic characteristics of individuals admitted with each vaccine preventable infection in 2017-20 and in 2020-21

	Neisseria meningitidis		Strep. Pneumoniae		Haemophilus influenza		Measles		Mumps	
	N (% of total)	N (% of total)	N (% of total)	N (% of total)	N (% of total)	N (% of total)	N (% of total)	N (% of total)	N (% of total)	N (% of total)
	2017-20	2020-21	2017-20	2020-21	2017-20	2020-21	2017-20	2020-21	2017-20	2020-21
**Total number:**	618	191	270	108	47	22	149	15	103	48
**Sex**										
Male	355 (57.4)	105 (55)	155 (57.4)	50 (46.3)	25 (53.2)	15 (68.2)	80 (53.7)	5 (33.3)	60 (58.3)	35 (72.9)
Female	260 (42.1)	90 (47.1)	115 (42.6)	60 (55.6)	25 (53.2)	5 (22.7)	70 (47)	10 (66.7)	40 (38.8)	10 (20.8)
**Age group**										
<1	170 (27.5)	70 (36.6)	95 (35.2)	50 (46.3)	20 (42.6)	5 (22.7)	45 (30.2)	0 (0)	5 (4.9)	5 (10.4)
1-4	255 (41.3)	70 (36.6)	115 (42.6)	40 (37)	20 (42.6)	15 (68.2)	75 (50.3)	10 (66.7)	35 (34)	20 (41.7)
5-9	130 (21)	30 (15.7)	40 (14.8)	10 (9.3)	10 (21.3)	0 (0)	15 (10.1)	5 (33.3)	35 (34)	15 (31.3)
10-14	60 (9.7)	25 (13.1)	20 (7.4)	5 (4.6)	0 (0)	0 (0)	15 (10.1)	0 (0)	25 (24.3)	10 (20.8)
**Region of residence**										
North East	45 (7.3)	10 (5.2)	10 (3.7)	15 (13.9)	0 (0)	0 (0)	5 (3.4)	0 (0)	10 (9.7)	0 (0)
North West	140 (22.7)	40 (20.9)	45 (16.7)	25 (23.1)	5 (10.6)	0 (0)	20 (13.4)	0 (0)	25 (24.3)	10 (20.8)
Yorkshire and Humber	100 (16.2)	60 (31.4)	60 (22.2)	10 (9.3)	5 (10.6)	0 (0)	20 (13.4)	0 (0)	10 (9.7)	5 (10.4)
East Midlands	45 (7.3)	15 (7.9)	20 (7.4)	5 (4.6)	5 (10.6)	0 (0)	5 (3.4)	5 (33.3)	5 (4.9)	0 (0)
West Midlands	100 (16.2)	20 (10.5)	25 (9.3)	15 (13.9)	0 (0)	5 (22.7)	20 (13.4)	0 (0)	10 (9.7)	5 (10.4)
East of England	45 (7.3)	10 (5.2)	20 (7.4)	5 (4.6)	5 (10.6)	0 (0)	10 (6.7)	0 (0)	10 (9.7)	10 (20.8)
London	60 (9.7)	15 (7.9)	35 (13)	15 (13.9)	10 (21.3)	0 (0)	50 (33.6)	5 (33.3)	15 (14.6)	10 (20.8)
South East	60 (9.7)	15 (7.9)	40 (14.8)	15 (13.9)	10 (21.3)	0 (0)	15 (10.1)	0 (0)	10 (9.7)	5 (10.4)
South West	25 (4)	10 (5.2)	20 (7.4)	5 (4.6)	5 (10.6)	5 (22.7)	10 (6.7)	0 (0)	5 (4.9)	0 (0)
**Deprivation quintile**										
1 (Most deprived)	225 (36.4)	65 (34)	70 (25.9)	35 (32.4)	15 (31.9)	5 (22.7)	60 (40.3)	5 (33.3)	35 (34)	15 (31.3)
2	130 (21)	45 (23.6)	65 (24.1)	25 (23.1)	10 (21.3)	0 (0)	35 (23.5)	5 (33.3)	20 (19.4)	5 (10.4)
3	95 (15.4)	20 (10.5)	45 (16.7)	25 (23.1)	10 (21.3)	5 (22.7)	20 (13.4)	0 (0)	15 (14.6)	5 (10.4)
4	90 (14.6)	35 (18.3)	55 (20.4)	20 (18.5)	5 (10.6)	5 (22.7)	15 (10.1)	0 (0)	15 (14.6)	15 (31.3)
5 (Least deprived)	75 (12.1)	25 (13.1)	40 (14.8)	5 (4.6)	10 (21.3)	10 (45.5)	10 (6.7)	0 (0)	15 (14.6)	5 (10.4)
**Ethnic category**										
White	515 (83.3)	175 (91.6)	200 (74.1)	85 (78.7)	35 (74.5)	10 (45.5)	100 (67.1)	10 (66.7)	75 (72.8)	30 (62.5)
Mixed	20 (3.2)	5 (2.6)	10 (3.7)	5 (4.6)	0 (0)	0 (0)	10 (6.7)	5 (33.3)	5 (4.9)	0 (0)
Asian	35 (5.7)	0 (0)	35 (13)	10 (9.3)	5 (10.6)	5 (22.7)	15 (10.1)	5 (33.3)	10 (9.7)	5 (10.4)
Black	15 (2.4)	5 (2.6)	10 (3.7)	5 (4.6)	5 (10.6)	0 (0)	10 (6.7)	0 (0)	5 (4.9)	5 (10.4)
Other	20 (3.2)	5 (2.6)	5 (1.9)	0 (0)	0 (0)	0 (0)	15 (10.1)	0 (0)	5 (4.9)	5 (10.4)
**Comorbidity**										
No known comorbidity	565 (91.4)	180 (94.2)	0 (0)	0 (0)	0 (0)	0 (0)	140 (94)	10 (66.7)	90 (87.4)	40 (83.3)
Asthma	30 (4.9)	0 (0)	0 (0)	0 (0)	0 (0)	0 (0)	5 (3.4)	0 (0)	10 (9.7)	0 (0)
Bronchopulmonary dysplasia	5 (0.8)	0 (0)	0 (0)	0 (0)	0 (0)	0 (0)	0 (0)	0 (0)	0 (0)	0 (0)
Extreme prematurity <28 weeks	5 (0.8)	0 (0)	0 (0)	0 (0)	0 (0)	0 (0)	0 (0)	0 (0)	0 (0)	0 (0)
Cystic fibrosis	0 (0)	0 (0)	0 (0)	0 (0)	0 (0)	0 (0)	0 (0)	0 (0)	0 (0)	0 (0)
Congenital cardiac disease	20 (3.2)	5 (2.6)	0 (0)	0 (0)	0 (0)	0 (0)	5 (3.4)	0 (0)	0 (0)	0 (0)
Bronchiectasis	0 (0)	0 (0)	0 (0)	0 (0)	0 (0)	0 (0)	0 (0)	0 (0)	0 (0)	0 (0)
Immunodeficiencies	0 (0)	0 (0)	0 (0)	0 (0)	0 (0)	0 (0)	0 (0)	5 (33.3)	0 (0)	0 (0)
Acute lymphoblastic leukaemia	0 (0)	0 (0)	0 (0)	0 (0)	0 (0)	0 (0)	0 (0)	0 (0)	0 (0)	0 (0)
Acute myeloid leukaemia	0 (0)	0 (0)	0 (0)	0 (0)	0 (0)	0 (0)	0 (0)	0 (0)	0 (0)	0 (0)

Subgroup numbers are rounded to the nearest 5, in accordance with NHS Digital reporting guidelines, to preclude deductive identification of individual patients where numbers are small

Supplementary Figure 1:
Percentage change in the number of individuals admitted,
from Mar 2017 - Feb 2020 (mean over 3 years) to Mar 2020 - Feb 2021, by hospital trust

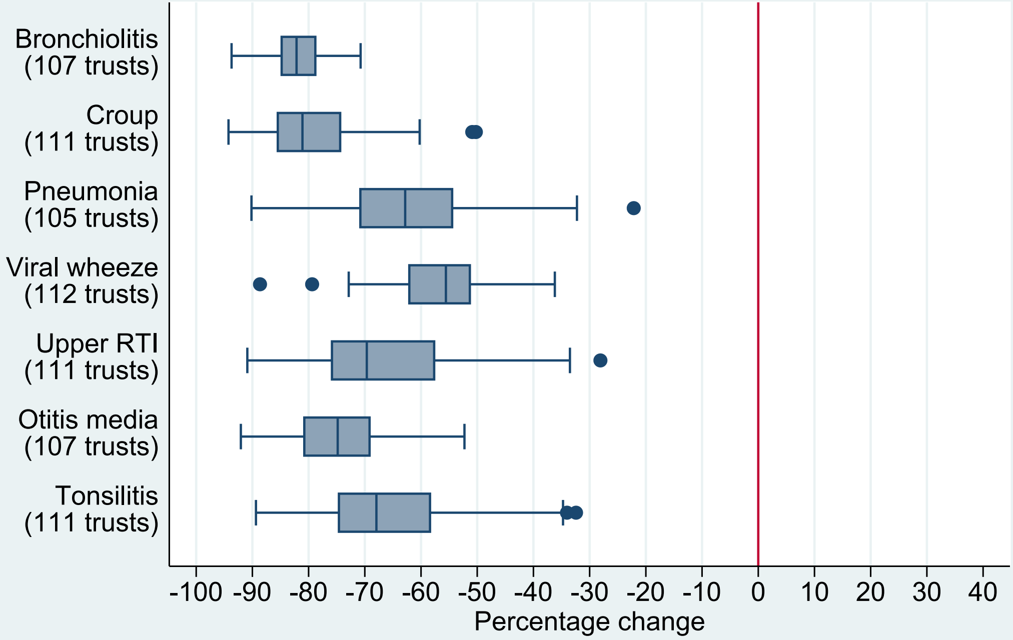

Supplementary Figure 2: Respiratory infections
Percentage change in the number of individuals admitted, from Mar 2017 - Feb 2020 (mean over 3 years) to Mar 2020 - Feb 2021

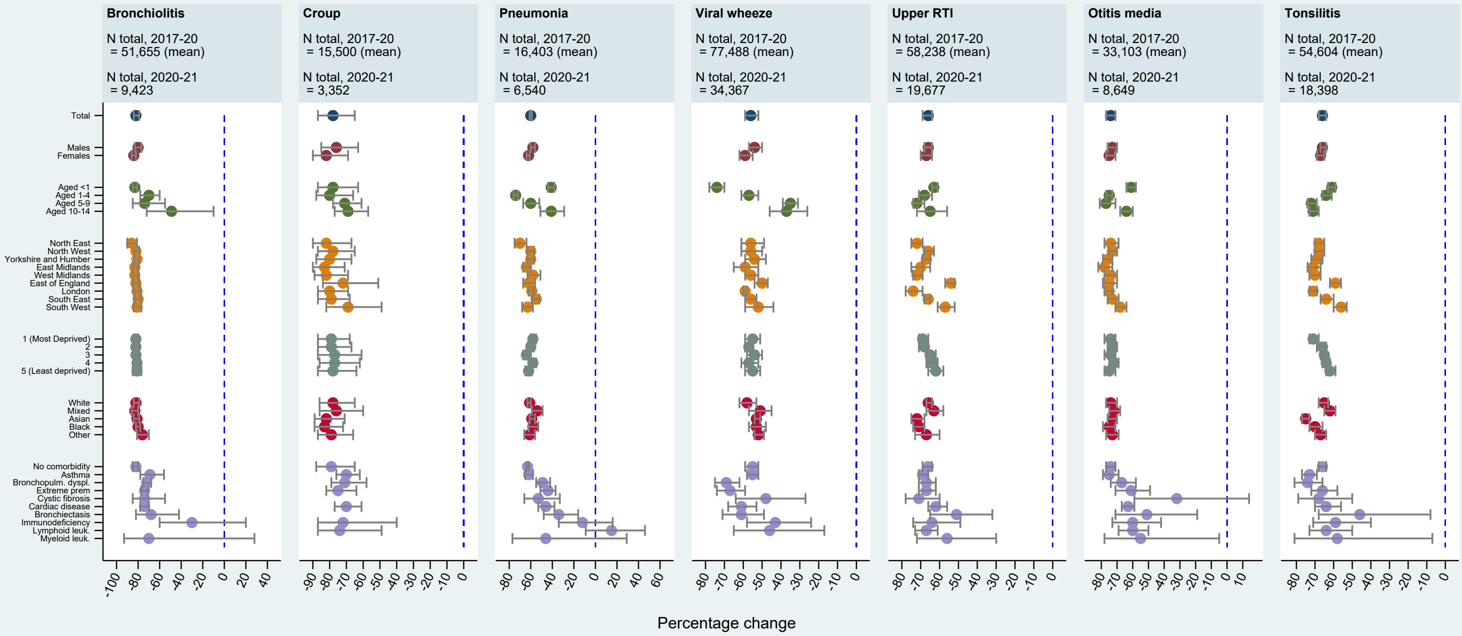

Counts <=5 supressed

Supplementary Figure 3: Invasive infections

Percentage change in the number of individuals admitted, from Mar 2017 - Feb 2020 (mean over 3 years) to Mar 2020 - Feb 2021

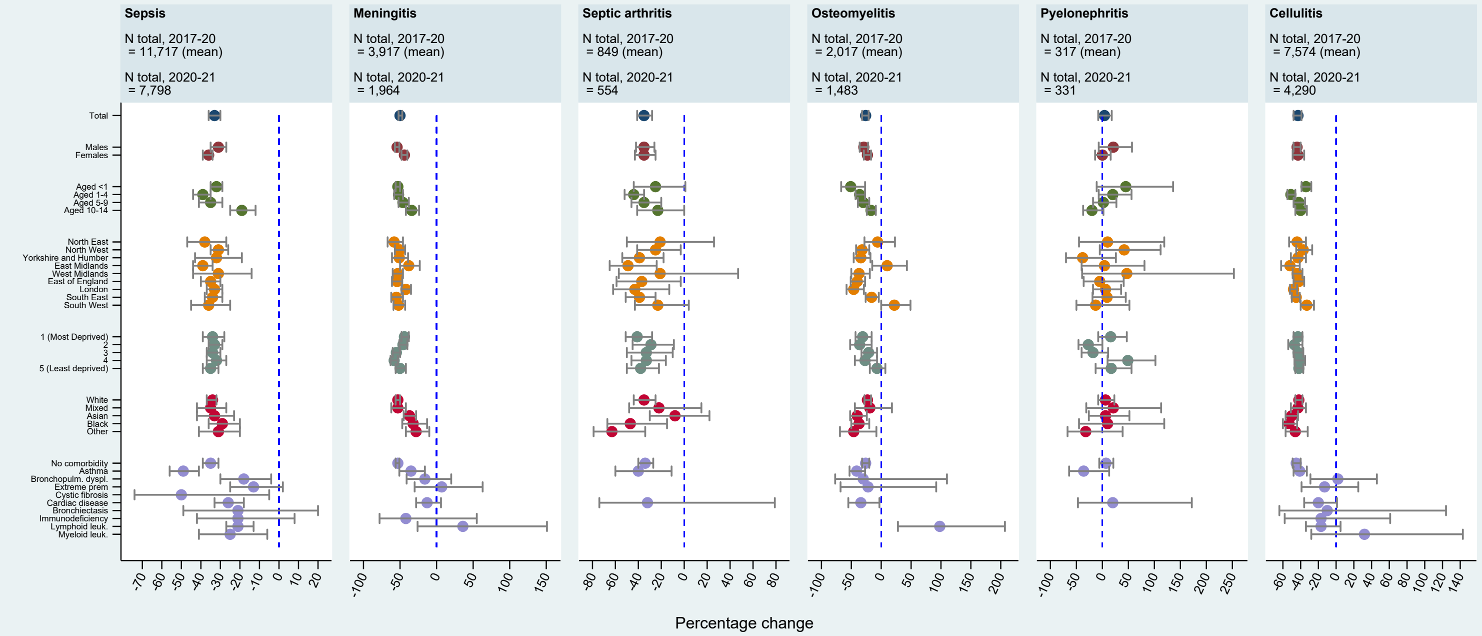

Counts <=5 supressed

Supplementary Figure 4: Vaccine preventable infections

Percentage change in the number of individuals admitted, from Mar 2017 - Feb 2020 (mean over 3 years) to Mar 2020 - Feb 2021

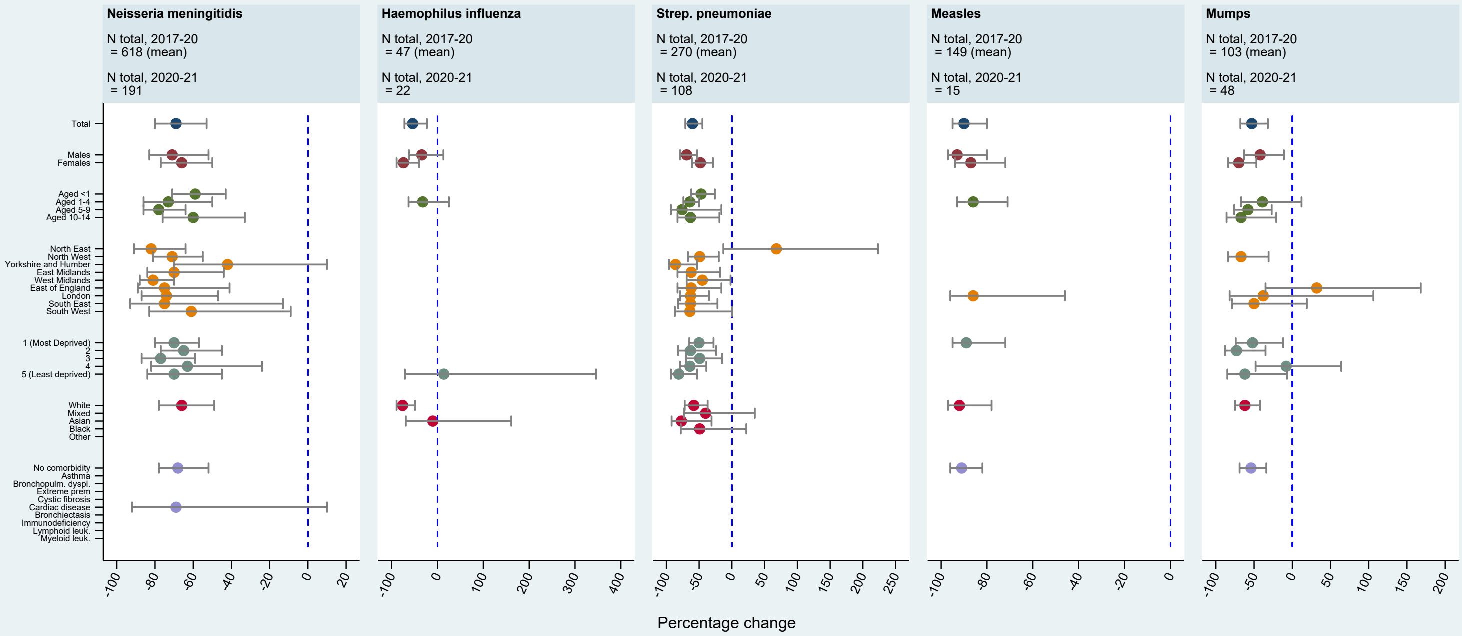

Counts <=5 supressed
